# EEG-Meta-Microstates: Towards a More Objective Use of Resting-State EEG Microstate Findings Across Studies

**DOI:** 10.1007/s10548-023-00993-6

**Published:** 2023-07-29

**Authors:** Thomas Koenig, Sarah Diezig, Sahana Nagabhushan Kalburgi, Elena Antonova, Fiorenzo Artoni, Lucie Brechet, Juliane Britz, Pierpaolo Croce, Anna Custo, Alena Damborská, Camila Deolindo, Markus Heinrichs, Tobias Kleinert, Zhen Liang, Michael M Murphy, Kyle Nash, Chrystopher Nehaniv, Bastian Schiller, Una Smailovic, Povilas Tarailis, Miralena Tomescu, Eren Toplutaş, Federica Vellante, Anthony Zanesco, Filippo Zappasodi, Qihong Zou, Christoph M Michel

**Affiliations:** 1 Translational Research Center, University Hospital of Psychiatry, University of Bern, Bern, Switzerland; 2 Department of Neurobiology, Care Sciences and Society, Center for Alzheimer Research, Division of Clinical Geriatrics, Karolinska Institutet, Huddinge, Sweden; 3 Children’s Hospital Los Angeles, The Saban Research Institute, Los Angeles, CA 90027 USA; 4 Division of Psychology, Department of Life Sciences, College of Health, Medicine and Life Sciences & Centre for Cognitive Neuroscience, Brunel University London, Kingston Lane, Uxbridge, UB8 3PH UK; 5 Human Neuron Lab, Faculty of Medicine, Department of Clinical Neurosciences, University of Geneva, Geneva, Switzerland; 6 Department of Basic Neurosciences, University of Geneva, Campus Biotech, 9 Chemin des Mines, Geneva, 1202 Switzerland; 7 Department of Psychology, University of Fribourg, Fribourg, Switzerland; 8 Department of Neurosciences, Imaging and Clinical Sciences, Behavioral Imaging and Neural Dynamics Center, Institute for Advanced Biomedical Technologies, “Gabriele d’Annunzio” University, Chieti, 66100 Italy; 9 Department of Nuclear Medicine, Geneva University Hospital (HUG), Geneva, Switzerland; 10 Department of Psychiatry, Faculty of Medicine, University Hospital Brno, Masaryk University, Brno, Czechia; 11 Center of Functionally Integrative Neuroscience, Aarhus University, Aarhus, Denmark; 12 Department of Psychology, Laboratory for Biological Psychology, Clinical Psychology and Psychotherapy, Albert-Ludwigs-University of Freiburg, Breisgau, Germany; 13 Department of Ergonomics, Leibniz Research Centre for Working Environment and Human Factors, Ardeystr. 67, Dortmund, 44139 Germany; 14 School of Biomedical Engineering, Medical School, Shenzhen University, Shenzhen, China; 15 Department of Psychiatry, Harvard Medical School, Boston, MA USA; 16 McLean Hospital, Belmont, MA USA; 17 Department of Psychology, University of Alberta, Edmonton, AB T6G 2E9 Canada; 18 Departments of Systems Design Engineering and Electrical & Computer Engineering, University of Waterloo, 200 University Avenue W, Waterloo, ON N2L 3G1 Canada; 19 Department of Clinical Neurophysiology, Karolinska University Hospital, Stockholm, Sweden; 20 Life Sciences Centre, Institute of Biosciences, Vilnius University, Vilnius, Lithuania; 21 CINETic Center, National University of Theatre and Film “I.L. Caragiale” Bucharest, Bucharest, Romania; 22 Faculty of Educational Sciences, Department of Psychology, University “Stefan cel Mare” of Suceava, Suceava, Romania; 23 Faculty of Psychology and Educational Sciences, Department of Cognitive Sciences, University of Bucharest, Bucharest, Romania; 24 Department of Neurology, Istanbul Eyupsultan Public Hospital, Istanbul, Turkey; 25 Program of Neuroscience Ph.D, Graduate School of Health Sciences, Istanbul Medipol University, Istanbul, Turkey; 26 Department of Psychology, University of Miami, Coral Gables, FL USA; 27 Center for MRI Research, Academy for Advanced Interdisciplinary Studies, Peking University, Beijing, 100871 China

**Keywords:** EEG, Resting-state, Microstates, Meta-analysis, Spatial similarity, Mental states, Functional brain states

## Abstract

Over the last decade, EEG resting-state microstate analysis has evolved from a niche existence to a widely used and well-accepted methodology. The rapidly increasing body of empirical findings started to yield overarching patterns of associations of biological and psychological states and traits with specific microstate classes. However, currently, this cross-referencing among apparently similar microstate classes of different studies is typically done by “eyeballing” of printed template maps by the individual authors, lacking a systematic procedure. To improve the reliability and validity of future findings, we present a tool to systematically collect the actual data of template maps from as many published studies as possible and present them in their entirety as a matrix of spatial similarity. The tool also allows importing novel template maps and systematically extracting the findings associated with specific microstate maps from ongoing or published studies. The tool also allows importing novel template maps and systematically extracting the findings associated with specific microstate maps in the literature. The analysis of 40 included sets of template maps indicated that: (i) there is a high degree of similarity of template maps across studies, (ii) similar template maps were associated with converging empirical findings, and (iii) representative meta-microstates can be extracted from the individual studies. We hope that this tool will be useful in coming to a more comprehensive, objective, and overarching representation of microstate findings.

## Introduction

The last decades of research into system-level dynamics of the human brain have recognized that spontaneous brain activity follows well-organized and replicable coactivation patterns. The basic tenet of neuroscience states that: (i) functional and mental states materialize as brain states of synchronous neural firing, (ii) a systematic inventory of such brain states can reliably be mapped onto an equally systematic inventory of functional and mental states. Following this, a vast body of empirical research has indeed shown that such spontaneously organizing brain resting states can reliably be identified and partly resemble task-elicited states (Smith et al. 2009), but see also (Davis et al. 2017). These states mirror in their variance the variance observed in human function and experience across healthy and pathological conditions (Castellanos et al. 2013).

While most research studies on resting-state networks to date have used fMRI, EEG scalp fields are an alternative means to non-invasively measure and compare human brain states. Such data yield temporally unfiltered, instantaneous, and spatially summated scalp-projected local neuro-electric potentials of the whole brain, thereby permitting tracking of the dynamics of brain functional state changes with millisecond resolution (Michel and Murray 2012).

Spontaneously occurring EEG scalp fields tend to aggregate into a relatively small number of prototypical spatial distributions, which permit the extraction of a confined set of functional brain states that replicate within and across individuals (Khanna et al. 2014; Koenig et al. 2002; Michel and Koenig 2018; Pascual-Marqui et al. 1995; Wackermann et al. 1993; Zanesco et al. 2020). This observation, made first in the eighties (Lehmann 1990; Lehmann et al. 1987), anticipated similar discoveries in resting state fMRI data (Biswal et al. 1995; Raichle et al. 2001). In addition, these scalp fields tend to remain stable for sub-second periods, which has made researchers conclude that brain information processing follows approximately stepwise dynamics, where each such step corresponds to a network of highly synchronized local nodes that facilitate the flow of information to nodes of other networks (Lehmann et al. 1987; Michel and Koenig 2018). These single steps of brain information processing have been called microstates (Lehmann et al. 1987). EEG microstates are thus a unique mean to quantify the dynamics of brain functional state changes in a time resolution compatible with the speed of human thought and information processing (Koenig et al. 2005; Lehmann et al. 1998; Wackermann et al. 1993). Over the last 50 years, a large body of empirical data has related the spatial configuration or topographies of these microstates and the temporal dynamics of these microstates to a broad range of functional and dysfunctional states of humans (Bréchet et al. 2020a; De Bock et al. 2020; Lehmann et al. 2010; Milz et al. 2016; Rieger et al. 2016; Tarailis et al. 2021, 2023).

However, for microstate research to thrive as an overall coherent research program (Lakatos 1978), it is necessary to find correspondences of specific microstate spatial distributions and their spatiotemporal dynamics with specific functional and mental states that generalize across all these individual studies. This implies that we must be able to identify similar prototypical microstate configurations across corresponding studies. So far, to ensure compatibility of findings, studies have addressed this problem by *a priori* choosing a reference set of normative microstate prototype maps, typically drawn from large cohorts of healthy subjects (Custo et al. 2017; Koenig et al. 2002), and labeling their newly obtained template maps by this common reference. Beyond this, the reference to these normative prototype maps (that were sometimes also called canonical microstate maps) has also been frequently used to justify the choice of the number of microstate classes by arguing that choosing the same number of classes as these canonical template maps is necessary to compare findings across studies. Using this approach, the results from new studies have been related to previously published studies using equal or similar labels even when there are topographical differences in the microstate maps (Michel and Koenig 2018).

While this approach has been somewhat successful in the past, it is not immune to criticism. Most importantly, the comparability of microstates across studies does not depend on the number of microstate classes employed being equal but on the actual topographical similarities of the microstate maps, which are supposed to represent similar underlying generators, brain functions, and mental states. A closer inspection of the four-class solution microstate maps of many studies that referenced their template maps to the “canonical four” maps of the first normative EEG microstate study (Koenig et al. 2002), however, reveals that there are considerable topographic variances in the template maps assigned to the same microstate class (Michel and Koenig 2018). This entails that findings attributed to the same microstate class may be associated with only very partially overlapping brain states. In addition, this approach limits the potential of the methodology to the capacity of such canonical templates to adequately represent the entire repertoire of brain states of interest. Apart from the rather obvious objection that a set of only four microstate classes, sampled with only 19 channels, may not be the most promising candidate for such an undertaking, it remains questionable if it is at all reasonable to aim for a “one size fits all” microstate template gold standard. Factors such as brain maturation, lesions, state of wakefulness, drug effects, mental diseases, or brain plasticity may lead to systematic changes in brain networks recruited under some circumstances which may generate topographically distinct microstates.

Other issues plague the universal use of the four “canonical microstates”. Due to a lack of alternatives, the similarity of microstate maps across studies has often been assessed by visual comparison of the topographies with published figures. This approach is inexact, prone to errors, and offers no quantitative description of similarities between microstate topographies. Another critical gap in the field is the inability to query literature based on spatial topographies of microstates instead of the labels associated with the microstates. This entails the risk that, eventually, interesting associations between studies remain undiscovered if the researchers fail to anticipate these associations between specific topographies, irrespective of class labels, and specifically search for them. And finally, to date, no studies have examined the commonality or spatial correlations of microstate template map configurations across studies. In our opinion, these issues justify an in-depth analysis of the topographic variability of microstate maps across studies and their associated inverse solutions.

The present project aims to overcome these issues by addressing the problem of comparing microstate map topographies and the associated findings across studies in a novel, quantitative manner. Instead of a rigid common classification of microstates that is purely based on labels, we propose to build a comprehensive database of microstate template map topographies from as many published studies as possible and augment this database with the empirical findings associated with these template map topographies. To do so, we have developed two user-friendly, MATLAB-based, interactive graphical user interface (GUI) applications through which the database can be accessed. These applications allows the users to: (i) add microstate template maps from their own data (using the MSTemplateEditor.mlapp), (ii) query the database to identify template maps from other studies that share a sufficiently high and quantifiable amount of variance with the template maps they have extracted in their data (using the MSTemplateExplorer.mlapp), and (iii) use the reported findings associated with the thereby identified template maps of other studies to guide the interpretation of their own results (using the MSTemplateExplorer.mlapp).

Compared to the currently employed approach of assigning and labeling newly found microstate template maps according to a single set of published “canonical” templates, the new approach has many advantages. First, this approach has the potential to identify cross-study microstate maps, which can serve as a more comprehensive reference for sorting and labeling microstate classes in future studies. This ensures that future studies do not bias the entire field towards a few privileged “canonical maps” that have been used as a reference for interpreting novel findings in most cases. Second, the similarity among the maps of the different studies can now be quantitatively expressed as the amount of shared variance in their spatial distribution, which is a good proxy of the shared variance in source space. Third, the novel method is far less sensitive to the number of classes identified in a new study: even if different studies have unequal numbers of microstate classes, the shared spatial variance is the most relevant factor that matters when associating the microstate template maps with results from other studies. Users may thus associate findings from other studies if the template maps of their and the referenced studies are sufficiently similar even if different numbers of microstates were identified. On the other side, researchers should refrain from doing so if these template maps are not sufficiently similar, even if the same number of classes were identified. Finally, traditional tools of literature research, such as PubMed or Web of Science, do not permit querying the literature using the spatial topography of a microstate class as a search criterion. This application allows users to search for findings associated with similar template maps and uncover interesting associations across the growing number of microstate studies.

The present paper presents the first version of the MATLAB-based application and demonstrates the analytical advantages of the novel approach proposed. The following methods section will describe how we collected and organized existing data and findings on EEG microstate templates, how the similarities among these template maps were computed and visualized, how one can extract empirical findings from the database, and how across study meta microstate maps were computed.

## Methods

### Application Information

**Fig. 1:**
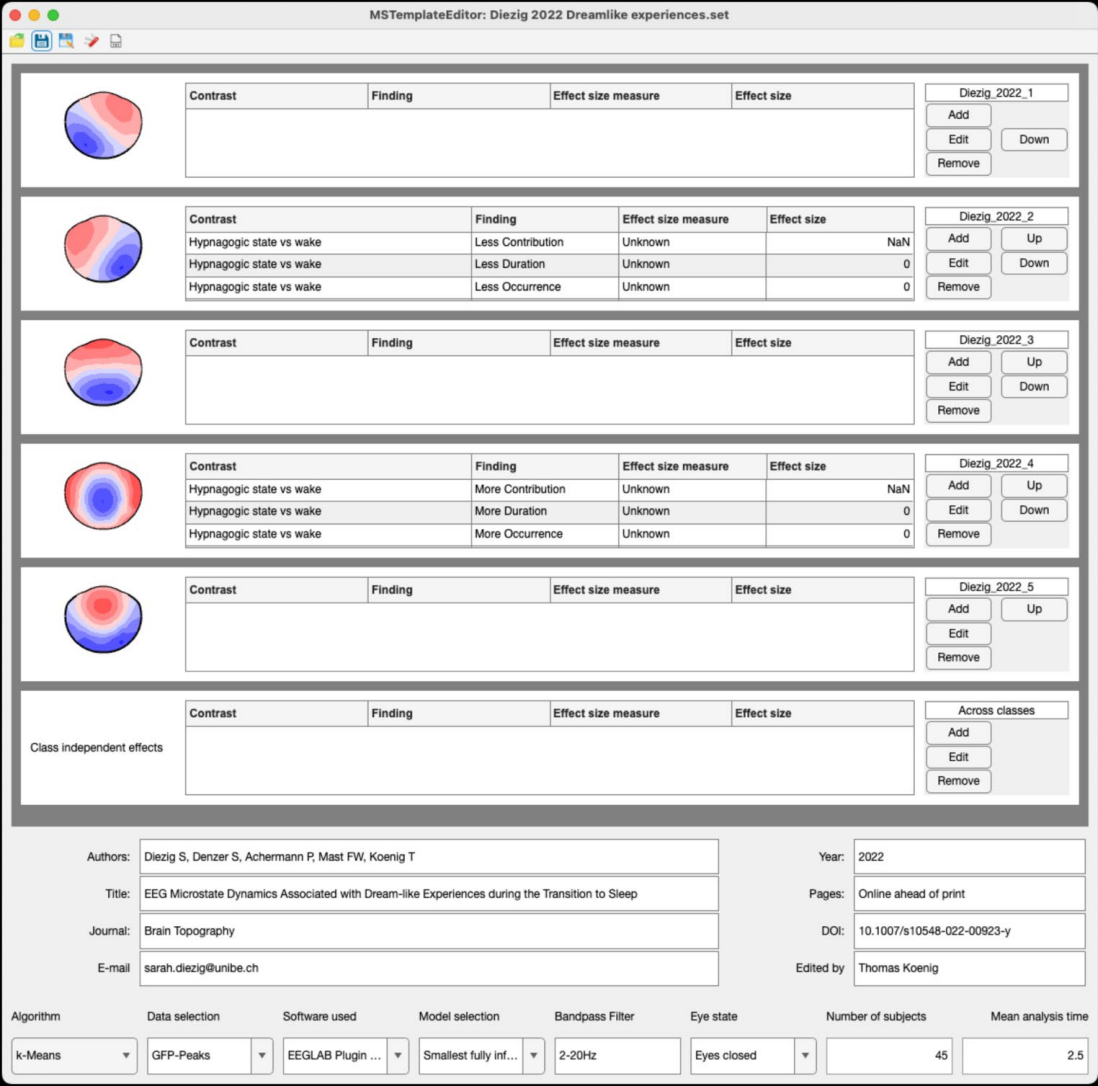
Screenshot of the MSTemplateEditor with the template maps and meta-data of the study by Diezig et al. 2022, on EEG microstates during the transition to sleep

The MATLAB applications and the template maps from published studies collected so far are available here: https://github.com/ThomasKoenigBern/MS-Template-Explorer. The applications are available as ready-to-use and standalone MATLAB apps and were developed and tested with MATLAB version 2022b. The applications have several functions:

- The MSTemplateEditor app allows users to add microstate template maps from their own data. A screenshot of the MSTemplateEditor with a sample dataset is shown in Fig. 1. To import new template maps, the MSTemplateEditor requires the template map data to be in a numeric and tabular format, along with the coordinates of the electrode positions.
- The MSTemplateExplorer has several built-in functions which are available as independent tabs.
  i. **Similarity Matrix** – This tab allows users to explore the topographical similarities of their maps with those of other studies in the database. To generate the similarity matrix, the amount of shared variance (i.e., the squared spatial correlation coefficient) among all available microstate template maps is computed pairwise. The obtained similarity matrix can then be used to compute a multidimensional scaling (MDS) to provide an intuitive visualization of the similarities and differences among template maps across various studies.
  ii. **MDS** - The MDS procedure assigns each template map to a point in a two- or three-dimensional space and arranges the positions of these points such that their pairwise distances optimally represent the similarities the similarity matrix gives. Topographically similar maps are thus represented at similar locations in this space, whereas topographically dissimilar maps are represented at different locations. The MDS display of the MSTemplateExplorer also allows users to select and plot individual template maps using the mouse cursor or the maps’ labels. Once a series of similar maps has been selected, the associated empirical findings are made available to the user in a tabular form in the “Findings” tab. The 3D view of the MDS display can also be rotated for visual exploration.
  iii. **Findings –** The tab allows users to systematically access the empirical findings associated with the template maps from published studies that are included in the database, which may allow for improved interpretation of results in future studies. The MSTemplateExplorer also permits the extraction of all the empirical findings across studies that are related to a selected meta-microstate map. All selected findings and the corresponding publications can be exported to an Excel spreadsheet. This grouping of study maps and their associated finding based on spatial similarity parallels the approach by Tarailis et al. (2023) but uses a fully data-driven procedure.
  iv. **Meta Clusters –** This tab allows for the computation and visualization of meta-maps, i.e., the spatial clusters of all the microstate template maps across studies. The MSTemplateExplorer also allows users to manually rearrange the sequence of the obtained meta-microstate maps, to backfit them to the template maps from individual studies in the MDS tab and extract all the associated findings for a given meta-map. Users can also export the obtained meta-maps in a format compatible with the microstate toolbox presented in this issue (Kleinert et al., n.d.) and use these maps directly for microstate feature extraction.

### Computational Information

#### Database Development

We contacted as many researchers as possible who have published on resting-state EEG microstates and asked them to send us the scalp field potential data of the mean microstate template maps they had identified in their published studies. Altogether, we collected 43 sets of template map data from 40 studies (Antonova et al. 2022; Artoni et al. 2022; Bréchet et al. 2020a; Britz et al. 2010; Croce et al. 2018, 2020, 2022; Custo et al. 2017; Damborská et al. 2019a, b; Deolindo et al. 2021; Diezig et al. 2022; Hanoglu et al. 2022; Hu et al. 2023; Kleinert et al. 2022; Koenig et al. 2002; Liu et al. 2020; Murphy et al. 2018; Musaeus et al. 2020; Nagabhushan Kalburgi et al. 2020; Nash et al. 2022; Notturno et al. 2023; Ricci et al. 2020, 2022; Schiller et al. 2019, 2020; Smailovic et al. 2019; Spring et al. 2017, 2018, 2022; Tarailis et al. 2021; Tomescu et al. 2018, 2022; Vellante et al. 2020; Zanesco et al. 2020, 2021a, b, c; Zappasodi et al. 2017, 2019). The obtained data were revised and converted to a common data format compatible with the EEGLAB microstate analysis toolbox presented in this issue (Kleinert et al., n.d.) using the MSTemplateEditor function specifically designed for this purpose. Only findings containing a contrast between groups and/or conditions or reporting inverse solutions were considered. Within each study, only grand-mean maps were entered unless there was a statistically corroborated difference in map topography. In addition to the microstate template maps from various studies, the meta-data of these template maps was also included in the database. The metadata consists of the most critical analysis parameters used in the study, information about the subjects’ groups and conditions at the time of data collection, publication data, and, most importantly, the findings associated with the temporal dynamics of each microstate class reported in the publication.

#### Similarity Matrix and Multidimensional Scaling

**Fig. 2:**
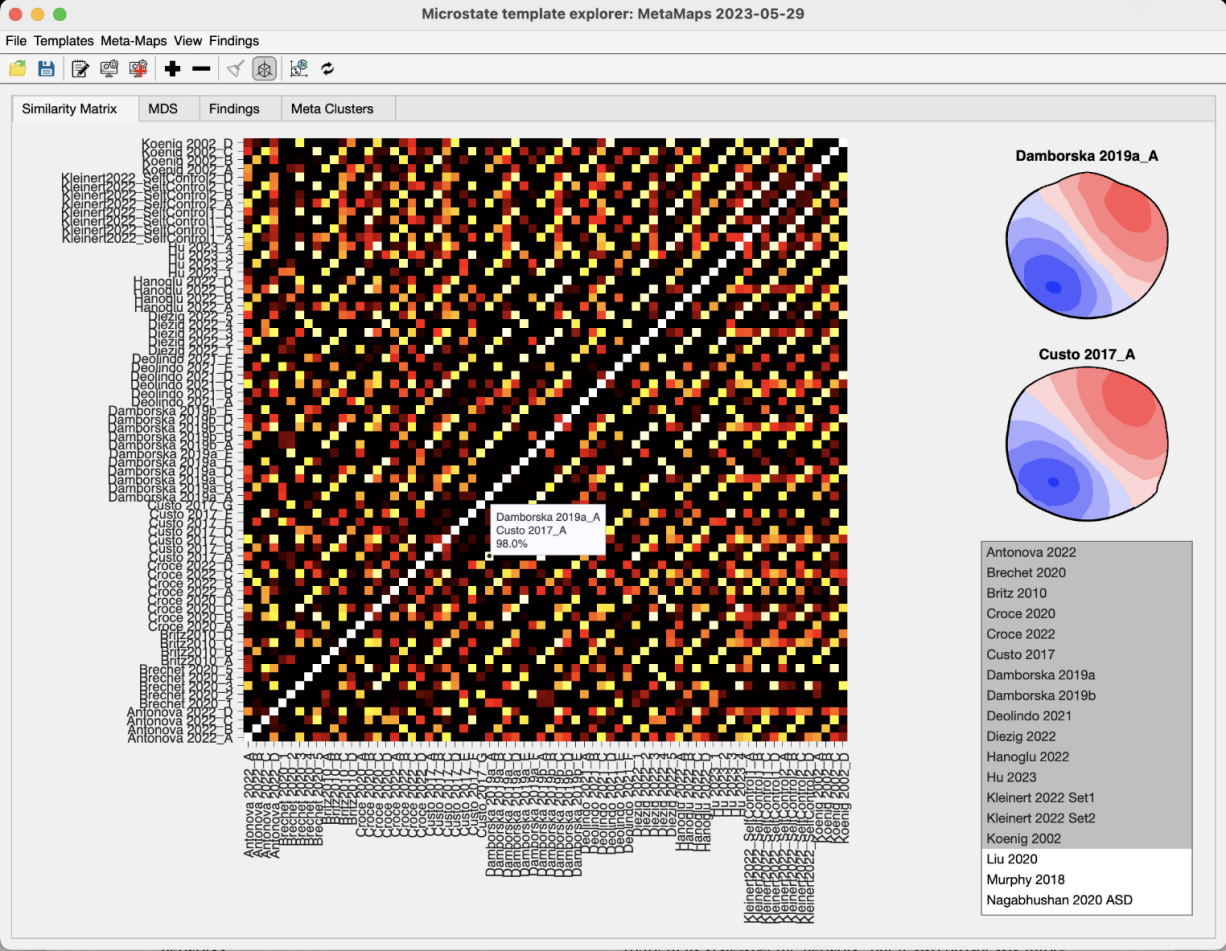
The similarity matrix obtained from 21 sets of template maps from different studies. Each column and row represent one template map of one study. Increasing brightness of the similarity matrix indicates increasing spatial similarity among pairs of maps. Note that many ‘lines’ of high similarity parallel the diagonal, suggesting that the obtained maps were similar and similarly ordered across many studies. A data tip can be used to read out the shared variance for any pair of maps that is of interest, and a click on an element of the similarity matrix plots the corresponding pairs of maps. The “+” and “-” buttons can be used to change the lower end of the color scale (lower cut-off value) of the similarity matrix, the upper end of the scale is always 100%. Note that for visual clarity, not all available studies have been selected for the display

Once the template maps of all included studies were processed and associated with their metadata, a similarity matrix was computed to quantify the similarity of microstate template maps across all maps of all studies. The visualization of these similarities in a subset of 21 studies is shown in Fig. 2. For the standard case where the two maps have different electrode montages, the MSTemplateExplorer spatially resampled the data to the electrode montage with fewer electrodes using spherical splines (Perrin et al. 1989) before the shared variance was computed. The similarities among microstate template maps were computed as the squared spatial correlation coefficient, which is identical to the shared variance. For the computation of the MDS, these values were recomputed to the dissimilarity value as defined in (Lehmann and Skrandies 1980) and used as input of Euclidean distances.

#### Across-study meta-microstate map Clusters

To generate the meta-microstate map clusters, the user needs to select a reasonably general electrode setup onto which all the template maps from the studies chosen are interpolated using spherical splines (Perrin et al. 1989). The maps of the individual studies are then weighted by the number of included subjects and submitted to the modified k-means algorithm (Pascual-Marqui et al. 1995), the standard approach for identification of within-subject template microstate maps, to identify the meta-maps. In the current implementation, the clustering is done for a range from 4 to 7 clusters, which spans the usual range of cluster numbers used in resting state EEG microstate studies. For demonstration purposes, the 5-cluster solution of the meta-microstate maps was used to backfit the template maps across all the studies included, and the empirical findings associated with one of the 5-class meta-microstate maps was extracted for demonstration.

#### Percent Overlap in Assignment of maps across Different Cluster Numbers

As we did not have sufficiently unequivocal criteria to select the number of meta-microstate maps and resorted to computing a series of solutions with different numbers of clusters, we investigated the quantitative effect of adding or removing microstate classes when fitting them to EEG resting-state data. Therefore, we conducted a small analysis on a sample dataset of resting state EEG of healthy subjects (Kleinert et al., n.d.) (https://osf.io/yqt7k/) to look at the commonality of the meta-microstate map assignment when using solutions with different numbers of classes. For this purpose, the different sets of meta-microstate maps we had obtained in the previous step were separately backfitted to the EEGs, yielding, for each number of meta-microstate maps, a time series of assignments. Based on these multiple assignments, we computed, for each combination of cluster numbers, the percent overlap in assignment for each combination of meta-microstate classes (see Fig. 7), yielding what we will call matrices of commonality of microstate assignment. This overlap in assignment between different numbers of clusters is informative about the changes in assignment to be expected when changing from a solution with a given number of classes to a solution with a different number of classes.

## Results

### Similarity Matrix

The similarity matrix obtained from the 39 included studies is shown in Fig. 2. The matrix shows clear “lines” of high map similarity that parallel the identity diagonal. This indicates that most studies have, as intended, arranged their microstate template maps in a common sequence. Furthermore, across studies, the similarities of corresponding microstate maps are often high, indicating good reproducibility of microstate maps across studies (this point has been made earlier, e.g., by Michel and Koenig 2018, or Tarailis et al. 2023). There are, however, also cases of microstate template maps that have rarely been described or that do not map well onto the topographies of the “canonical four” microstate classes, producing some gaps and shifts in this overall pattern (e.g., for the maps of Deolindo et al. 2021 that were recorded in actively flying helicopter pilots).

### Multidimensional Scaling

**Fig. 3:**
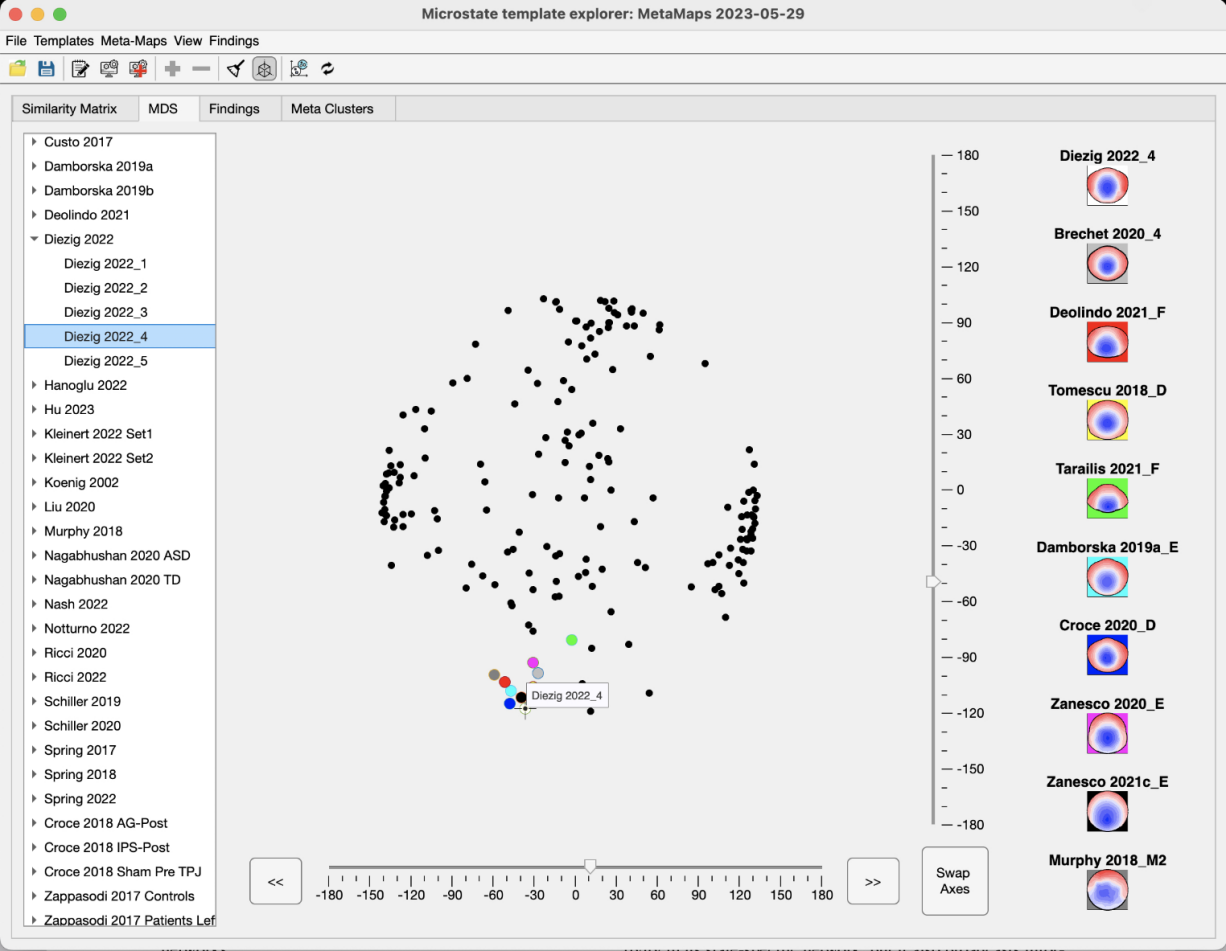
MDS display of the similarity matrix shown after a few maps have been selected and displayed. The point representing a particular map can also be identified using the list on the left side. Note again the striking visual similarity of the selected maps

The MDS display obtained from the similarity matrix is shown in Fig. 3. It is apparent that most of the microstate maps of the different studies aggregate in a series of clouds, confirming their replicability across studies. In the figure, a set of points in a region of such a cloud has been selected to show the associated map topographies and studies, which allows the user to interactively identify microstate maps from other studies that are similar to maps identified in her/his own dataset(s). To extract the exact similarities among the selected maps, users can switch to the Similarity Matrix tab and select only the chosen subset of studies (panel in the lower right part of the display).

### Database of Empirical Findings

**Fig. 4:**
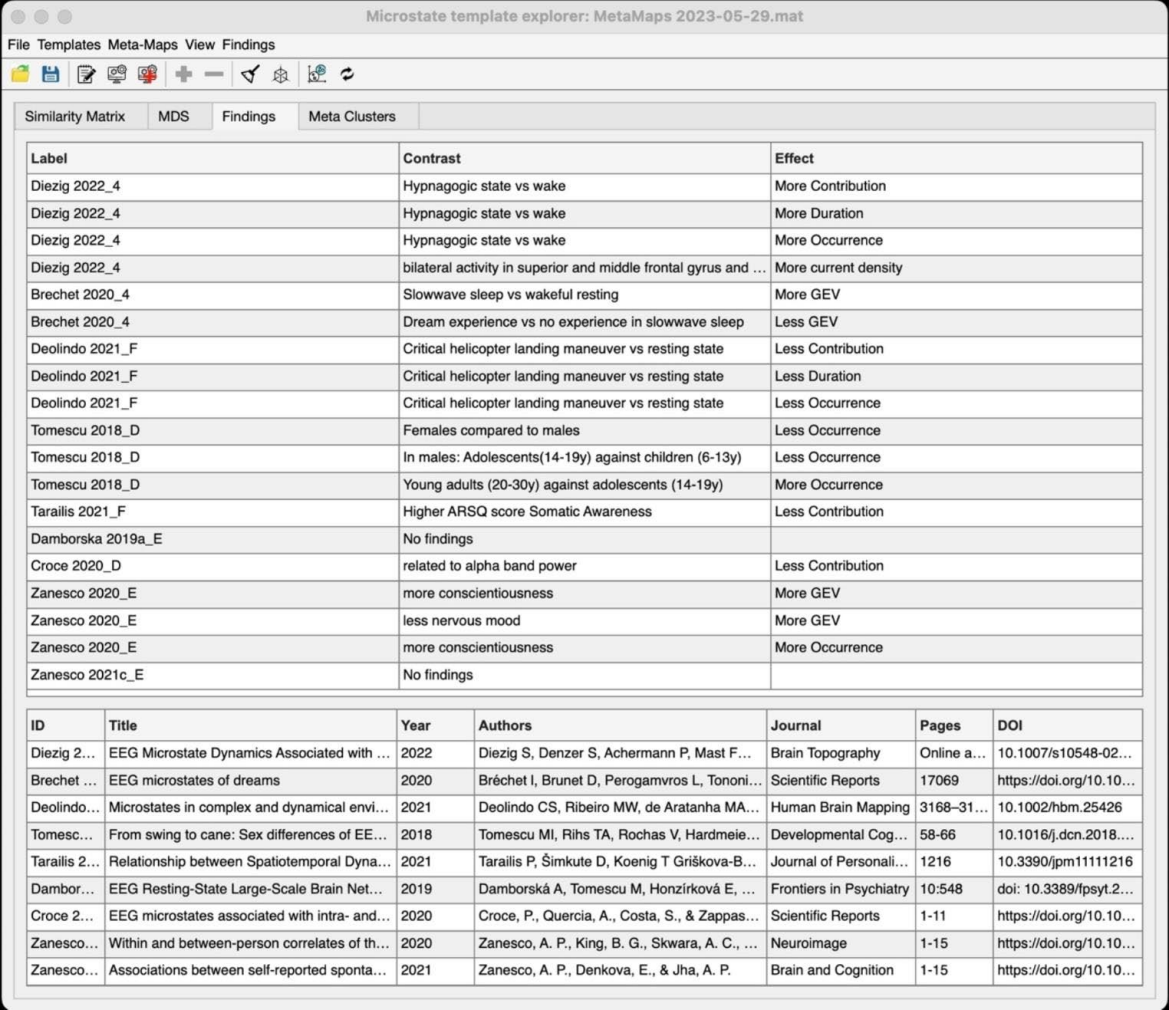
The complete list of findings associated with the maps selected in Fig. 3. Note that there are major inconsistencies in the labelling of these maps despite their spatial similarity. At the same time, there are consistent empirical findings: The list suggests, e.g., that with increasing presence of the selected map(s), there is a progression from a state of high concentration (Deolindo et al. 2021), through increasing hypnagogic experiences during rest (Diezig et al. 2022) to a state of first dreaming and then dreamless sleep (Bréchet et al. 2020b)

The findings associated with the topographies of maps selected above (Fig. 3) were explored. The presently available collection of the empirical findings associated with the selected maps is shown in Fig. 4. Users can, therefore, directly explore the association of “their” template maps with the findings/conclusions made in previous publications based on the topographical similarity of the template maps.

### Meta-microstate maps, Similarities in Microstate Topographies across Classes, and Empirical Findings associated with meta-microstate maps

**Fig. 5:**
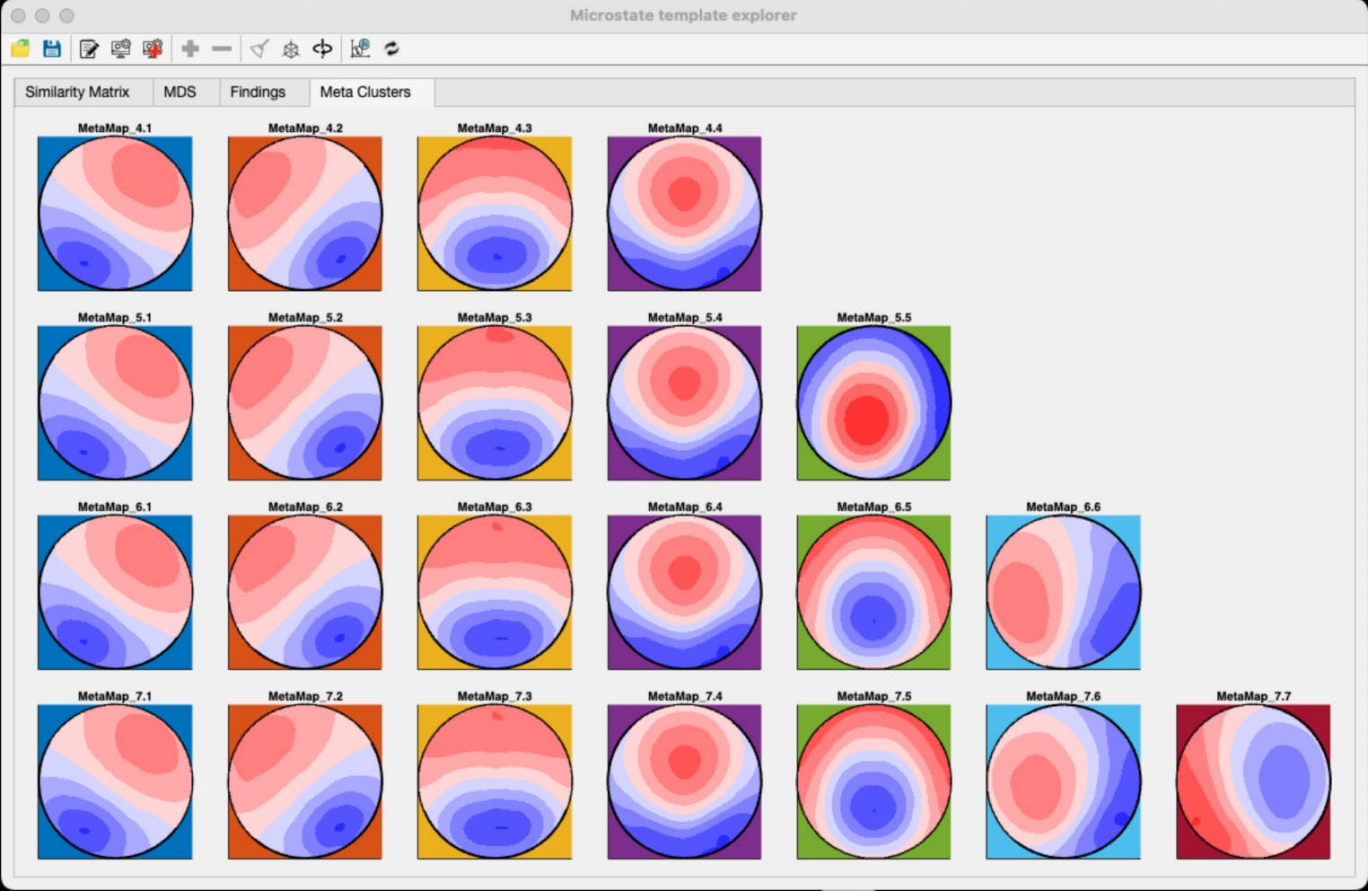
“Meta-microstate template maps” obtained after clustering across the template maps of all included studies. Solutions ranging from 4 to 7 classes are shown. Note the high correspondence of maps across the different solutions

The resulting meta-microstate maps obtained from the included studies are shown in Fig. 5. Unsurprisingly, the obtained maps are very similar to the well-known prototypical microstate maps (Custo et al. 2017; Koenig et al. 2002). Interestingly, however, moving from a solution with a given cluster number to a solution with one more cluster produced very little change in the topographies of the obtained classes, but instead added a class with a new topography. The newly computed meta-maps show the “canonical four” microstate topographies in the 4-class solution, and these same “canonical four” microstate topographies can indeed be seen across all the solutions of meta-microstate maps.

**Fig. 6:**
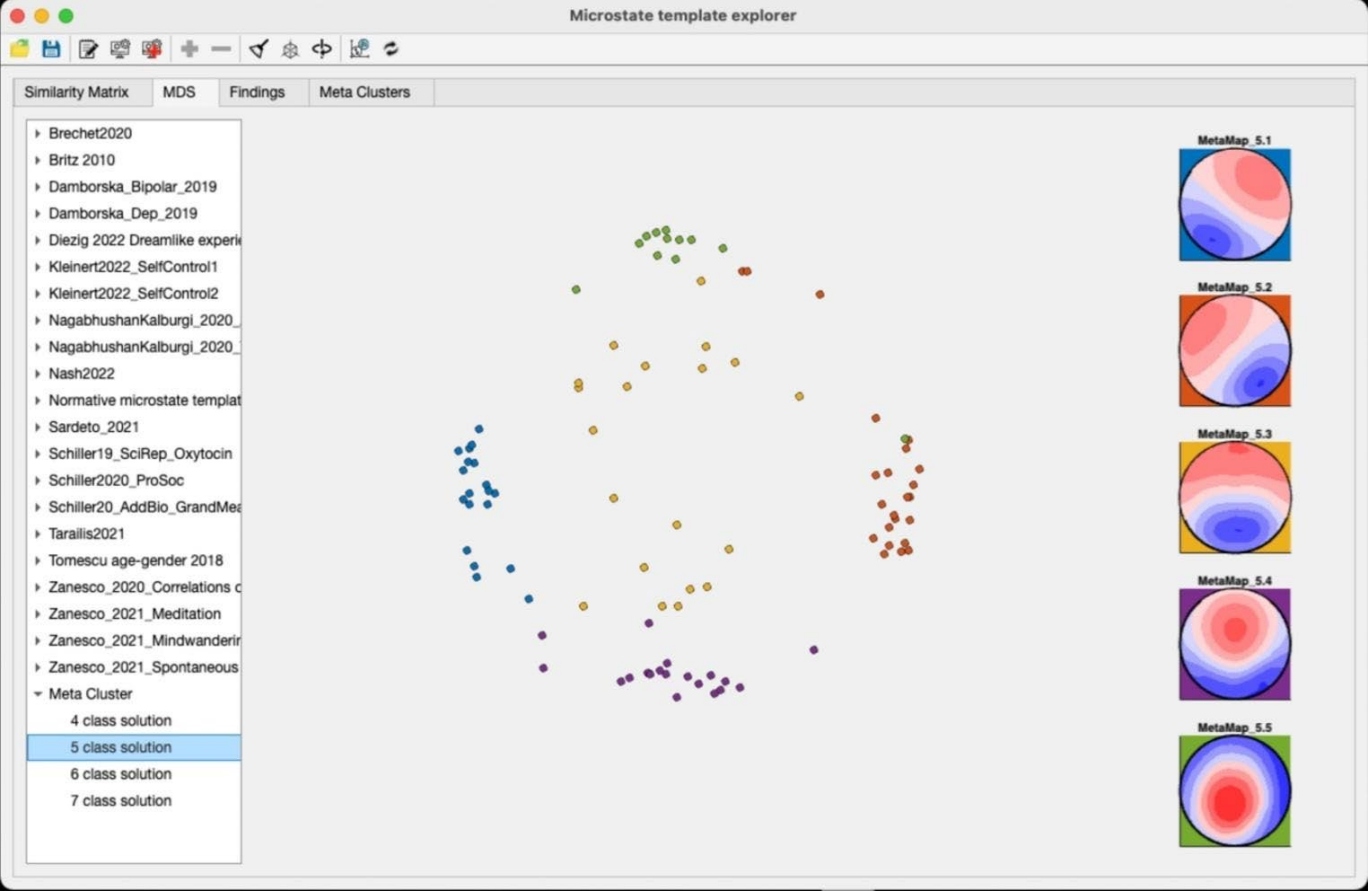
The back-fitting of the 5 class meta-microstate template maps to the template maps of the individual studies. Note that because all microstate maps are scaled to equal GFP (i.e., unit vector length), they are technically on a n-dimensional hypersphere, that is then reduced in dimensionality by the multidimensional scaling. The display represents this hypersphere in just two dimensions, yielding the (false) impression that the clusters at the horizon of the sphere are more compact. The MSTemplateExporer allows switching to a 3D representation, which permits a more appropriate visual impression

The backfitting of the 5-class meta-microstate map solution of the meta-maps to the template maps of the individual studies is shown in Fig. 6. The assignment confirms the clear cluster structure of the data across studies (and is visually even more convincing in the 3D display also available in the app). An example of the findings associated with a particular meta-microstate class is shown in Table 1.

**Table 1:** All database findings associated with the 5th microstate class of the 5-class meta-map solution. Note that the labels of the microstate maps correspond to the labeling in the database and not necessarily to the reference list of this article

Label	Contrast	Effect
Antonova 2022_C	No findings	
Brechet 2020_4	Slowwave sleep vs. wakeful resting	More GEV
Brechet 2020_4	Dream experience vs. no experience in slowwave sleep	Less GEV
Croce 2020_D	related to alpha band power	Less Contribution
Croce 2022_D	isometric contraction task against rest	More Duration
Croce 2022_D	isometric contraction task against rest	More Occurrence
Custo 2017_G	Right inferior parietal lobe extending to the superior temporal gyrus	More current density
Custo 2017_G	Cerebellum	More current density
Damborska 2019a_E	No findings	
Damborska 2019b_E	No findings	
Deolindo 2021_F	Critical helicopter landing maneuver vs. resting state	Less Contribution
Deolindo 2021_F	Critical helicopter landing maneuver vs. resting state	Less Duration
Deolindo 2021_F	Critical helicopter landing maneuver vs. resting state	Less Occurrence
Diezig 2022_4	Hypnagogic state vs. wake	More Contribution
Diezig 2022_4	Hypnagogic state vs. wake	More Duration
Diezig 2022_4	Hypnagogic state vs. wake	More Occurrence
Diezig 2022_4	bilateral activity in superior and middle frontal gyrus and precuneus	More current density
Murphy 2018_M2	No findings	
Tarailis 2021_F	Higher ARSQ score Somatic Awareness	Less Contribution
Tomescu 2018_D	Females compared to males	Less Occurrence
Tomescu 2018_D	In males: Adolescents(14-19y) against children (6-13y)	Less Occurrence
Tomescu 2018_D	Young adults (20-30y) against adolescents (14-19y)	More Occurrence
Vellante 2020_D	No findings	
Zanesco 2020_E	more conscientiousness	More GEV
Zanesco 2020_E	less nervous mood	More GEV
Zanesco 2020_E	more conscientiousness	More Occurrence
Zanesco 2021 Meditation_E	No findings	
Zanesco 2021 Meditation_F	No findings	
Zanesco 2021b_E	mindwandering against on-task	Less GEV
Zanesco 2021b_E	mindwandering against on-task	Less Occurrence
Zanesco 2021b_E	mindwandering against on-task	Less Contribution
Zanesco 2021c_E	No findings	

### Percent Overlap in the Assignment of maps across Different Cluster Numbers

**Fig. 7:**
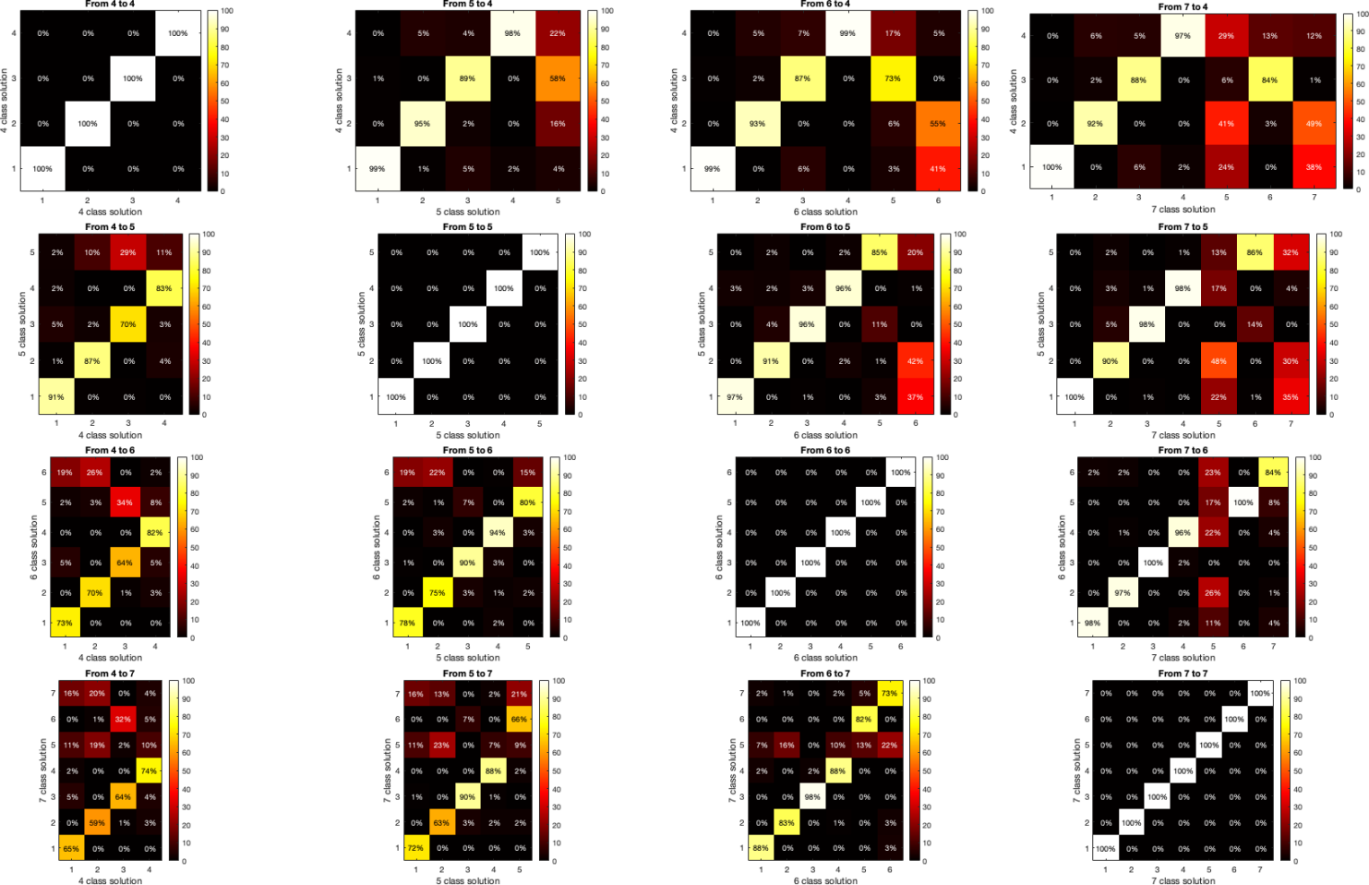
Commonality (percent overlap) of microstate assignment among meta-microstate maps with different numbers of classes. Each graph represents the commonalities of the assignment of microstates observed when changing between solutions with different numbers of classes of the meta-microstate maps. The color-coded bins of the graphs indicate the percentage of time points that are common for the given assignment. The data is normalized to make the sum of commonalities 100% over columns. For example, the second graph from the left in the first row indicates that 99% of all time points assigned to class 1 in the 4-class solution were assigned to class 1 in the 5-class solution, and 1% of all time points assigned to class 1 in the 4-class solution were assigned to class 3 in the 5-class solution

The matrices of commonality of microstate assignment across classes for the sample dataset are shown in Fig. 7. These matrices generally show very high values on the diagonal, where the commonality of the spatially most similar meta-microstate maps is represented. This overlap in assignment between different solutions is informative about the changes in assignment to be expected when changing from a solution with a given number of classes to a solution with a different number of classes. Our results indicate that in the analyzed data, changing from a solution with a given number of classes to a solution with one more or one less class had little effect on the microstate assignment for all but one class.

## Discussion

This project aims to build a comprehensive database of published resting state microstate template maps and their empirical findings and to develop a user-friendly interface to query topographical similarities across past studies and provide users with a platform to compare the topographies of their own studies with published ones. An analysis of a total of 39 studies we had available at the time of the writing of this article showed a high commonality of microstate template map configurations across studies. This was true despite significant differences in analytical parameters, experimental setups, specific strategies, experimental questions, conditions, and groups. This overall high replicability of microstate configuration across studies is also reflected in the representative sets of ‘meta-microstate maps’ obtained by clustering across the template maps of the different studies. Topographically similar microstate template maps are assumed to represent similar active sources in the brain. As these sources can be assumed to constitute similar functional states, the empirical findings associated with these topographically similar template maps can be identified more objectively and systematically. The MSTemplateExplorer allows users to extract data-driven associations between empirical findings across studies, and the potential of this approach is likely to expand as more published template maps from independent studies are added.

Authors of past and future studies using the microstate approach are encouraged to submit their template maps using our GUI-based tool, MSTemplateEditor. By having an increasingly complete database of EEG-microstate template map topographies, we can change the status quo of the ‘eyeball’ approach for labeling microstate template map classes across studies. Using a more objective, data-driven index of template map similarities across a hopefully increasingly comprehensive collection of studies, we not only eliminate an element of arbitrariness in the rapidly growing field of EEG microstate analysis but also provide opportunities to examine interesting but previously overlooked associations between studies. As an example, findings related to the microstate templates maps collection shown in Figs. 3 and 4 not only show a systematic and continuous progression from a highly attentive state (Deolindo et al. 2021) to drowsiness and hypnagogic mentation (Diezig et al. 2022) to dreamless sleep (Bréchet et al. 2020a), but also to somatic awareness (Tarailis et al. 2021), which is something typically lost during decreased wakefulness.

Additionally, this approach provides a more objective means to relate microstate findings across studies, irrespective of the number of classes, which also partly addresses the still controversial issue of choosing an appropriate number of microstate clusters. In the past, it has often been argued that the number of classes should be selected such that the analysis is compatible with previous results, which explains the predominance of studies with four classes. However, from the theoretical point of view, having the same number of clusters does not necessarily imply that the topographies of template maps are comparable across studies. The identified maps may differ substantially even with the same number of clusters as seen in microstate class C in Fig. 3, in Michel and Koenig 2018. On the other hand, it is a necessary and sufficient condition that template maps share a high amount of topographical similarity to conclude that they share a high amount of source activity and, thus, presumably serve similar functions independent of the number of microstate classes used in the analysis. ‘Good’ choices of microstate class numbers may therefore be understood in the long run as choices that yield converging evidence across studies.

In this context, analyzing the commonality of microstate assignment across the different sets of meta-microstate maps (Fig. 7) further de-emphasizes the importance of finding a presumably correct number of classes to account for the data. Across the different sets of meta-microstate template maps, the assignment of the analyzed EEG data to the maps of the different sets of meta-microstates was highly stable for all but one or two microstate classes (Fig. 7). This indicates that also the typical class-wise microstate parameters such as microstate duration, microstate occurrence, or percent time covered are robust against changes in the number of classes used in many cases, and particularly for the ‘canonical four’ microstate classes. The impact of the yet-to-be-conclusively-solved problem of choosing the number of microstate classes may thus only have a limited impact on the field of EEG microstate analysis.

In conclusion, we hope that the present effort and the novel tools provided herein extend the methodological foundation for objectivity and comprehensiveness in the field of EEG resting state microstate analysis. The success of this project will depend on the willingness of the authors of past and future studies to provide their microstate template maps and on journals and reviewers to encourage such a policy. In return, the findings of studies that provide their microstate template maps are more likely to be examined in a wider context and expanded upon by future studies, thereby increasing their impact.
